# Functional Sub-Circuits of the Olfactory System Viewed from the Olfactory Bulb and the Olfactory Tubercle

**DOI:** 10.3389/fnana.2017.00033

**Published:** 2017-04-11

**Authors:** Masahiro Yamaguchi

**Affiliations:** Department of Physiology, Kochi Medical School, Kochi UniversityKochi, Japan

**Keywords:** odor identity, odor value, adult neurogenesis, centrifugal inputs, functional domain, motivated behavior

## Abstract

Understanding of the olfactory neural circuits has progressed beyond analysis of how odor information from the external environment is processed in the brain. While spatially-organized sub-circuits were found to exist up to the olfactory bulb (OB), the arrangement in the olfactory cortex (OC), especially in its representative piriform cortex (PC), appears diffuse and dispersed. An emerging view is that the activity of OC neurons may not simply encode odor identity but rather encode plastic odor information such as odor value. Although many studies support this notion, odor value can be either positive or negative, and the existence of sub-circuits corresponding to individual value types is not well explored. To address this question, I introduce here two olfactory areas other than the PC, OB and olfactory tubercle (OT) whose analysis may facilitate understanding of functional sub-circuits related to different odor values. Peripheral and centrifugal inputs to the OB are considered to relate to odor identity and odor value, respectively and centrifugal inputs to the OB potentially represent different odor values during different behavioral periods. The OT has spatially-segregated functional domains related to distinct motivated and hedonic behaviors. Thus, the OT provides a good starting point from which functional sub-circuits across various olfactory regions can be traced. Further analysis across wide areas of the olfactory system will likely reveal the functional sub-circuits that link odor identity with distinct odor values and direct distinct odor-induced motivated and hedonic behaviors.

## Association of Odor Identity and Odor Value in the Olfactory Cortex

Animals utilize sensory systems to understand the environment in which they live. A straightforward question for understanding sensory neural circuits is how sensory inputs are processed in the circuits. In answering this question, our understanding of olfactory neural circuits has been remarkably advanced by the identification of odorant receptors, axonal convergence of olfactory sensory neurons expressing a given odorant receptor, and the resulting formation of odor maps in the olfactory bulb (OB; Mori and Sakano, 2011; Dalton and Lomvardas, 2015).

Further analyses of the OC, the higher cortical center of the olfactory system, indicate that odor information processing in the OC is complex. The piriform cortex (PC) is spatially the widest area among various areas of the OC and has been supposed to be the functional representative of the OC. In contrast to the topographically-organized projection of olfactory sensory neurons to the OB, axonal projection of OB principal neurons to the PC is diffuse and dispersed (Ghosh et al., 2011; Sosulski et al., 2011; Igarashi et al., 2012). Neuronal activity in response to a given odor appears to be not spatially arranged but rather widely distributed throughout the PC (Stettler and Axel, 2009). Thus, odor maps in the OB appear to be discarded in the PC, a characteristic which hampers understanding of how odor information is processed in the higher-order cortical regions.

The anatomical organization of the OC is quite different from the sensory cortices of other modalities. The OC is not a six-layered neocortex but a three-layered paleocortex. The OC belongs to the limbic system and forms massive reciprocal connections with various brain areas, including the amygdala and prefrontal cortex (PFC; Shipley and Ennis, 1996; Haberly, 2001; Illig, 2005; Mori, 2014). The OC is thus regarded as an association cortex which integrates odor inputs and other neuronal activities within the brain. In fact, the activity of pyramidal cells in the PC depends mainly on association fiber inputs rather than peripheral inputs from the OB (Poo and Isaacson, 2011).

An emerging view is that OC neurons might not simply encode odor identity; rather, OC neuron activity is highly plastic and dependent on the circumstantial situation, including learning and memory, thereby encoding higher-order information such as odor value. The ensemble activity of PC neurons is altered in correlation with odor association learning and reversal learning (Calu et al., 2007; Roesch et al., 2007; Chapuis and Wilson, 2011). Information on odor identity and odor value is related to the temporal firing pattern of the PC neurons (Gire et al., 2013). The PC receives massive inputs from the lateral amygdala, and optogenetic stimulation of these inputs modulates the odor-induced activity of PC neurons (Sadrian and Wilson, 2015). These observations are consistent with the notion that the major function of PC neurons is to associate odor information with a particular value.

Even if this is the case, however, it remains totally unknown whether the association of odor information with a particular value is conducted by a particular population of PC neurons. Odors can be endowed with positive value as well as negative value, and the existence of sub-circuits contributing to individual value types has not been well explored. In addressing this question, while the PC is the representative of the OC its direct analysis might rather be difficult, because it is very spatially wide and communicates with various brain regions. Here, therefore, I introduce two olfactory regions other than the PC whose analysis may facilitate understanding of the possible existence of functional sub-circuits which encompass wide olfactory areas, including the PC. These are the OB and the olfactory tubercle (OT), which I discuss in the following sections.

## Olfactory Bulb Integrates Peripheral Odor Inputs and Central Activity of Higher Cortical Regions

**Figure 1:**
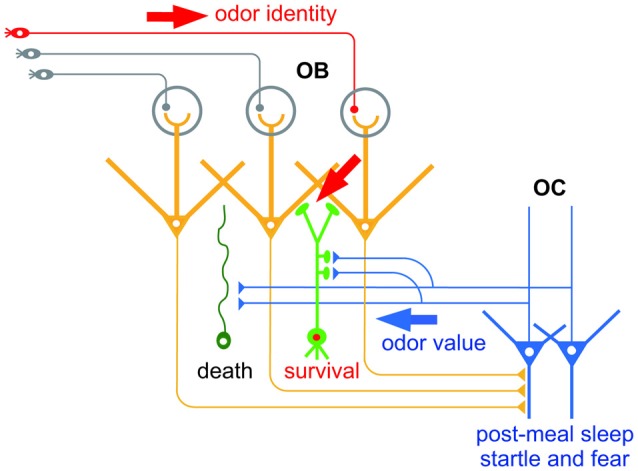
**Hypothetical scheme of the selection of new olfactory bulb (OB) neurons by odor value-related centrifugal inputs from the olfactory cortex (OC).** New OB neurons (green) receive peripheral inputs (red arrow) which convey information of odor identity. New neurons also receive centrifugal inputs from the OC (blue arrow) which promote selection of new OB neurons for survival or death. The central inputs presumably convey information of odor value, while inputs during post-meal sleep period and during startle and fear responses may convey distinct odor value information.

As the first relay of olfactory information processing, the OB receives odor information from the external environment. Odor input is transferred from olfactory sensory neurons in the olfactory epithelium to mitral/tufted cells, the principal projection neurons in the OB, through synapses in the glomerular neuropils (Shepherd et al., 2004). In addition, the OB receives central information via the OC. Pyramidal cells in the OC send glutamatergic centrifugal axonal projections to the OB (de Olmos et al., 1978; Haberly and Price, 1978a; Davis and Macrides, 1981; Manabe et al., 2011; Figure 1). Importantly, the centrifugal axons mainly project onto local inhibitory interneurons in the OB, deep layer-located granule cells (GCs) and superficial layer-located periglomerular cells (PGCs), all of which modulate the activity of mitral/tufted cells (Luskin and Price, 1983; Shepherd et al., 2004).

The centrifugal inputs from the OC have a strong impact on OB function. These inputs modulate mitral/tufted cell activity in the OB: when activated, they potentiate the inhibition of odor-evoked mitral cell activity by inhibitory interneurons (Boyd et al., 2012; Markopoulos et al., 2012). This raises the question of what information is conveyed by the centrifugal inputs—in particular, do they feedback specific information such as odor value to the OB and modulate mitral/tufted cell activity to ensure a suitable feedback information-dependent response?

The contribution of centrifugal inputs to structural reorganization of the OB neural circuit hints at the information they convey. An outstanding characteristic of the olfactory system is that neurogenesis continues throughout life (Lepousez et al., 2013). New neurons generated in the subventricular zone migrate rostrally to the OB and become interneurons such as GCs and PGCs. Similarly to the case of new neurons in the embryonic period, new GCs and PGCs generated in adulthood are selected for survival or death, a process which ensures the fine tuning of functional circuits. This selection occurs mostly within the OB circuit, and excess new GCs and PGCs are eliminated from the circuit by apoptotic mechanisms. Intriguingly, selection of new GCs occurs during specific behaviors, and is promoted by the centrifugal inputs from the OC during the behaviors (Figure 1). For example, elimination of new GCs is enhanced during the sleep period after eating, and is dependent on the centrifugal inputs from the OC during post-meal sleep (Yokoyama et al., 2011; Yamaguchi et al., 2013; Komano-Inoue et al., 2014). GC elimination is also enhanced by the delivery of an electrical foot shock, depending on the centrifugal inputs from the OC during startle and fearful responses (Komano-Inoue et al., 2015).

The contribution of centrifugal inputs to the structural reorganization that occurs during at least two different behaviors provides an opportunity to reveal the information conveyed by the centrifugal inputs. Because food is a typical reward for animals, centrifugal inputs after food eating might represent a positive value. On the other hand, because electrical shock is a strong punishment for animals, centrifugal inputs during and after electrical shock might represent a negative value. This notion fits with the observation that glutamatergic centrifugal projection to the OB regulates food intake behavior under the control of endocannabinoid signaling (Soria-Gómez et al., 2014). Given that the OC receives synaptic inputs from the amygdala and PFC (Haberly, 2001; Illig, 2005; Mori, 2014), the activity of OC neurons might be entrained to a positive or negative value via the inputs from these brain areas. Simultaneous analysis of the amygdala and PFC might reveal whether the PC integrates odor identity and odor value.

A related question in considering functional sub-circuits concerns the spatial organization of the centrifugal inputs. It is not yet known whether specific value information originates in specific populations of PC neurons, or whether specific value information is transmitted to specific populations of OB interneurons and modulates the activity of specific populations of mitral/tufted cells. An anatomical study showed that the boutons of centrifugal axons of individual PC neurons were distributed in a patchy but not diffuse pattern in the OB (Matsutani, 2010). The use of retrograde transsynaptic viral tracers showed that PC neurons that send centrifugal projection were spatially clustered in the PC (Padmanabhan et al., 2016). On the other hand, a functional study showed that centrifugal inputs tuned to different odors were diffusely distributed across glomerular maps in the OB (Boyd et al., 2015). While further studies are required, analysis of centrifugal inputs in the OB and its tracing back to the OC and beyond will unravel the functional sub-circuits in the olfactory neural circuit.

## The Olfactory Tubercle Has Spatially-Segregated Functional Domains Related to Distinct Motivated and Hedonic Behaviors

**Figure 2:**
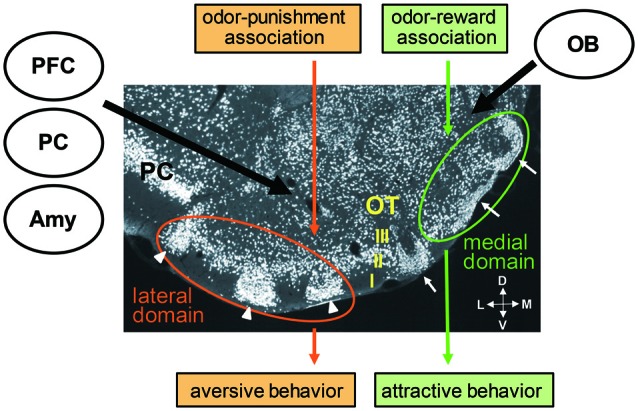
**Cytoarchitecture of the olfactory tubercle (OT) and its putative functional domains representing distinct odor-induced motivated behaviors.** A coronal section of the OT immunostained with anti-NeuN (a neuronal marker protein) antibody shows its three-layered structure (layers I, II and III) and involvement of cell-dense structures named cap compartments (white arrowheads) and Islands of Calleja (white arrows). An odor associated with reward activates the anteromedial domain of the OT and induces attractive behavior. An odor associated with punishment activates the lateral domain of the OT and induces aversive behavior. Individual OT domains receive inputs from the OB, amygdala (Amy), prefrontal cortex (PFC) and other OC areas, such as the piriform cortex (PC). M, medial; L, lateral; V, ventral; D, dorsal.

By definition, the OC is the brain region which receives the direct projection of OB principal neurons. The OC consists of various areas other than the PC, including the anterior olfactory nucleus, OT, nucleus of LOT, cortical amygdala and entorhinal cortex (Luskin and Price, 1983). Among these areas, the OT is unique in that it contains GABAergic medium spiny neurons as principal neurons and receives massive dopaminergic inputs from the ventral tegmental area in the midbrain. Thus, the OT also belongs to the ventral striatum, together with the nucleus accumbens (Heimer, 1978; Millhouse and Heimer, 1984; Ikemoto, 2007). While the OT basically has a three-layered cortex-like structure (layers I, II and III), it also contains cell-dense structures named cap compartments and Islands of Calleja (Fallon et al., 1978; Millhouse and Heimer, 1984; Figure 2). Cap compartments consist of small-sized medium spiny neurons and are distributed at the surface of the lateral portion of the OT. The Islands of Calleja consist of granule cells and are distributed across wide areas of the OT.

The ventral striatum is known to direct motivated behaviors and hedonic liking (Berridge and Kringelbach, 2015). Self-administration of cocaine or amphetamine in the ventral striatum facilitates hedonic self-administration (Ikemoto, 2003; Ikemoto et al., 2005; Shin et al., 2010). Given its dual attribution to the OC and ventral striatum, the OT is regarded as a candidate structure which links odor cues to motivated and hedonic behaviors (Wesson and Wilson, 2011). Recent functional studies support this notion. Electrical OT stimulation alters odor preference behavior (Fitzgerald et al., 2014), and lesions or DREADD-induced silencing of the OT abolishes the attraction of female mice to male chemosignals (Agustín-Pavón et al., 2014; DiBenedictis et al., 2015). Electrophysiological studies showed that the OT neurons encode odor valence and discriminate the type and magnitude of reward (Gadziola et al., 2015; Gadziola and Wesson, 2016).

Sub-regional segregation of function in the ventral striatum has been identified. For example, microinjections of an AMPA/kainate receptor antagonist or GABA_A_ receptor agonist in the anterior medial shell of the nucleus accumbens elicited appetitive eating behavior, whereas the same microinjections in the posterior medial shell induced fearful defensive treading (Reynolds and Berridge, 2002, 2003). Self-administration of cocaine or amphetamine was most strongly potentiated when these drugs were injected in the anteromedial part of the nucleus accumbens or OT, and was not potentiated on injection to the posterolateral nucleus accumbens or OT (Ikemoto, 2003; Ikemoto et al., 2005; Shin et al., 2010). Based on this background, the possible involvement of the different areas of the OT on different odor-cued motivated behaviors was examined. Findings showed that when an odor was associated with a food reward and the odor induced attractive behavior in the learned mice, the medium spiny neurons in the layer II of the anteromedial domain of the OT was activated. In contrast, when the same odor was associated with electrical foot shock punishment and the odor induced aversive behavior in the learned mice, the medium spiny neurons in the layer II as well as in the cap compartments of the lateral domain of the OT was activated (Murata et al., 2015). The medial OT domain of female mice was selectively activated by exposure to male-derived odor (DiBenedictis et al., 2015). These observations suggest the existence of distinct functional domains in the OT which direct distinct odor-cued motivated and hedonic behaviors (Figure 2). Further, the unique cytoarchitecture of the OT with cortical structures, cap compartments and Islands of Calleja raises the possibility that each structure plays a specific role in odor-cued behaviors.

The OT receives massive axonal projection from various cortical regions, including other areas of the OC (Haberly and Price, 1978a,b; Neville and Haberly, 2004; Santiago and Shammah-Lagnado, 2004; Narikiyo et al., 2014), orbitofrontal cortex, agranular insular cortex and medial PFC (Berendse et al., 1992; Figure 2). In addition, the OT receives axonal projection from the posteromedial cortical amygdala, a representative of the vomeronasal cortex that receives pheromonal signals from the accessory OB (Gutiérrez-Castellanos et al., 2014). This projection from the vemeronasal cortex occurs dominantly to the anteromedial OT including the Islands of Calleja. Thus the OT receives both odorous and pheromonal signals from main and accessory olfactory pathways. The OT therefore seems to occupy an ideal position to integrate many kinds of information, including odor identity and odor value, and to direct different types of olfactory motivated responses. Further analysis of the connectivity of individual functional OT domains with other brain regions will likely reveal the functional sub-circuits in the olfactory system.

## Conclusions

Understanding the functional olfactory circuit starts with an analysis of how odor inputs from the external environment are transferred and processed in higher-order olfactory neural circuits. In addition, recent analyses have begun to focus on the circuit mechanisms that encode odor values and conduct odor-induced motivated and hedonic behaviors. The OB provides a good platform for understanding the link between odor inputs and odor value. The peripheral and central inputs to the OB are considered to relate to odor identity and odor value, respectively, and the existence of spatially-organized odor maps in the OB may help to dissect the possible spatial organization of odor value-based centrifugal inputs. The OT is considered to possess functional domains related to distinct motivated and hedonic behaviors, and to provide a good starting point from which functional sub-circuits across various olfactory regions can be traced.

While the anatomical and functional organization of the PC appears diffuse and dispersed, recent studies suggest heterogeneity in PC neurons. Output neurons from the PC to different areas of the orbitofrontal cortex were differently distributed in the PC along the dorsal-ventral axis (Chen et al., 2014). PC neurons activated in response to attractive odor cues and in response to aversive odor cues were distributed differently along the dorsal-ventral axis (Murata et al., 2015). Molecular analysis showed that some genes were expressed in subpopulations of PC neurons that were spatially segregated in distinct layers of the PC and connected with different target areas (Diodato et al., 2016). Further molecular, anatomical and functional analyses across wide areas of the olfactory system would reveal the functional sub-circuits that link odor identity with particular odor values and direct particular odor-induced motivated and hedonic behaviors.

## Author Contributions

MY wrote the manuscript.

## Conflict of Interest Statement

The author declares that the research was conducted in the absence of any commercial or financial relationships that could be construed as a potential conflict of interest.
